# Phosphorylated fragile X mental retardation protein at serine 499, is reduced in cerebellar vermis and superior frontal cortex of subjects with autism: implications for fragile X mental retardation protein-metabotropic glutamate receptor 5 signaling

**DOI:** 10.1186/2040-2392-4-41

**Published:** 2013-11-01

**Authors:** Øyvind G Rustan, Timothy D Folsom, Mahtab K Yousefi, S Hossein Fatemi

**Affiliations:** 1 Department of Psychiatry, Division of Neuroscience Research, University of Minnesota Medical School, 420 Delaware St SE, MMC 392, Minneapolis, MN 55455, USA; 2 Department of Pharmacology, University of Minnesota Medical School, 310 Delaware St SE, MMC 392, Minneapolis, MN 55455, USA; 3 Department of Neuroscience, University of Minnesota Medical School, 321 Delaware St SE, MMC 392, Minneapolis, MN 55455, USA

**Keywords:** mGluR5, FMRP, Cerebellar vermis, Superior frontal cortex, Phosphorylation of FMRP

## Abstract

Lohith et al. (Mol Autism 4:15, 2013) recently identified increased metabotropic glutamate receptor 5 (mGluR5) expression in the frontal cortex (FC) of subjects with fragile X syndrome. These results are consistent with postmortem findings in cerebellar vermis and FC of subjects with autism (Fatemi and Folsom, Mol Autism 2:6, 2011; Fatemi et al. Anat Rec 294:1635–1645, 2011), suggesting that increased mGluR5 signaling is common to multiple autism spectrum disorders. Increased mGluR5 signaling may be associated with reduced phosphorylation of fragile X mental retardation protein (FMRP), which could result in the inactivation of this protein. In the current study, we report on reduced expression of phosphorylated FMRP in cerebellar vermis of adults and children with autism and in FC of adults with autism.

## Findings

We have read with great interest the recent article by Lohith *et al*. [1] regarding increased expression of metabotropic glutamate receptor 5 (mGluR5) in the frontal cortex of individuals with fragile X syndrome (FXS). The results are consistent with our published work of increased expression of mGluR5 in the superior frontal cortex of children with autism [2]. Moreover, we have also demonstrated increased mGluR5 expression in the cerebellar vermis of children with autism [3]. Taken together, our data and those of Lohith *et al*. [1] suggest that increased brain expression of mGluR5 may be a specific marker of autism spectrum disorders. In contrast, we have identified reduced expression of mGluR5 in the brains of subjects with schizophrenia and bipolar disorder [4].

Increased mGluR5 expression in autism and FXS is associated with reduced or absent expression of fragile X mental retardation protein (FMRP) [5]. We have shown reduced FMRP expression in the cerebellar vermis and superior frontal cortex of individuals with autism [2,3] and from the lateral cerebellum and superior frontal cortex of subjects with schizophrenia, bipolar disorder, and major depression [4,6]. Additionally, levels of several targets of FMRP including ras-related C3 botulinum toxin substrate 1 (RAC1), homer 1, striatal-enriched protein tyrosine phosphatase (STEP), and amyloid beta A4 precursor protein (APP) are also altered significantly in subjects with autism [7] pointing to involvement of mGluR5-FMRP signaling abnormalities in autism. FMRP has been found to colocalize with stalled, translationally inactive polyribosomes when phosphorylated at serine 499, whereas dephosphorylated FMRP associates with actively translating ribosomes [8]. Thus, phosphorylated FMRP is seen as a translational repressor, while dephosphorylation of FMRP, mediated by mGluR signaling, may lead to derepression of protein translation.

**Figure 1:**
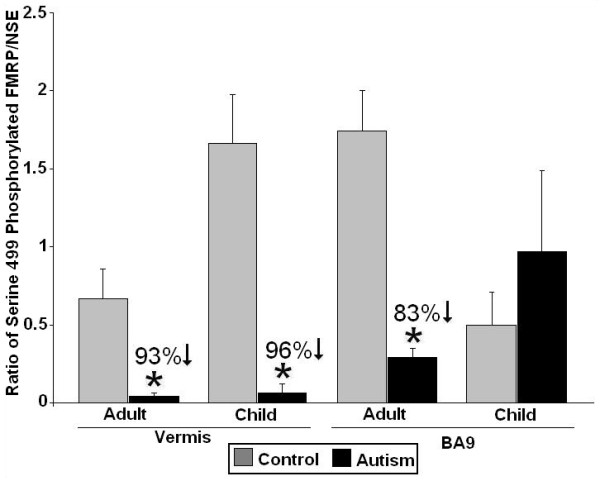
**Ratios of serine 499 phosphorylated Fragile X mental retardation protein (FMRP)/neuronal specific enolase (NSE) in the cerebellar vermis and Brodman area 9 (BA9) of subjects with autism versus matched controls.** Significant reductions were found in subjects with autism in the vermis of adults (*P* <0.012) and children ( *P* <0.0018), and in BA9 in adults (*P* <0.0001).

We have recently completed a preliminary study of serine 499 phosphorylated FMRP protein levels in the cerebellar vermis in adults (n = 5 controls and 5 adults with autism) and children (n = 3 controls and 4 children with autism), and in the superior frontal cortex in adults (n = 6 controls and 10 adults with autism) and children (n = 6 controls and 8 children with autism). All values were normalized against neuronal specific enolase (NSE) and data were expressed as ratios of phosphorylated FMRP/NSE. We found significant reductions in phosphorylated FMRP/NSE in the vermis of adults and children with autism when compared with controls (Figure 1). There was also a significant reduction in phosphorylated FMRP in the Brodman area 9 (BA9) in adults with autism, whereas there was no significant change in the BA9 of children (Figure 1). Age, gender, and postmortem interval (PMI) were examined as possible confounders. In those cases where the relationship between confounding variables and phosphorylated FMRP showed moderate or greater effect sizes (for example, *r* >0.3) we used analysis of covariance (ANCOVA) to co-vary their effects. In none of these cases were significant differences between controls and subjects with autism in phosphorylated FMRP changed by the presence of these covariates. Our new finding of a reduction in phosphorylated FMRP in the cerebellar vermis of children with autism may be associated with increased activity of mGluR5, which could result in dephosphorylation of FMRP, and its subsequent ubiquitination and degradation [9]. Current basic science reports showing abnormalities in FMRP-mGluR5 signaling and their targets [1-3,7] support the usefulness of new novel treatments in autism spectrum disorders.

## Abbreviations

ANCOVA: Analysis of covariance; APP: Amyloid beta A4 precursor protein; BA9: Brodman area 9; FMRP: Fragile X mental retardation protein; FXS: fragile X syndrome; mGluR5: Metabotropic glutamate receptor 5; NSE: Neuronal specific enolase; RAC1: Ras-related C3 botulinum toxin substrate 1; STEP: Striatal-enriched protein tyrosine phosphatase.

## Competing interests

The authors declare that they have no competing interests. SH Fatemi has several patents on the use of Reelin as a diagnostic marker for neuropsychiatric disorders but has not derived any financial gains from these patents.

## Authors’ contributions

SHF conceived of the study, participated in its design, supervised conduct of all experiments, and contributed to the drafting of the manuscript. OGR, MKY performed western blotting experiments. TDF performed western blotting experiments and contributed to the drafting of the manuscript. All authors read and approved the final manuscript.
